# Challenging Case of Anaplastic Thyroid Cancer With Unusual Clinical and Histological Features: A Diagnostic Dilemma With Undifferentiated Pleomorphic Sarcoma

**DOI:** 10.7759/cureus.53840

**Published:** 2024-02-08

**Authors:** Ali Alkhlaifat, Laith Albudour, Mohammad Buwaitel, Ra'ad Alomari, Alia Hajjat, Khaled Helael, Faris Abu Za’nouneh

**Affiliations:** 1 Department of Surgery, Royal Medical Services, Amman, JOR; 2 Department of Pathology, Royal Medical Services, Amman, JOR; 3 Department of Internal Medicine, Jordan University of Science and Technology, Irbid, JOR

**Keywords:** tumour markers, immunohistochemical stain, thyroid histology, undifferentiated pleomorphic thyroid sarcoma, anaplastic thyroid cancer

## Abstract

Thyroid cancer is a common malignancy worldwide, and its incidence is increasing rapidly, especially in women. In the majority of cases, it presents solely with a palpable neck swelling. Less commonly, the disease manifests with symptoms of advanced stages, such as superior vena cava (SVC) obstruction and indications of recurrent laryngeal nerve invasion.

Anaplastic thyroid cancer is a rare variant of thyroid cancer and is considered to have one of the poorest prognoses, and its diagnosis and treatment are challenging. On the other hand, undifferentiated pleomorphic sarcoma is a differential diagnosis with many clinical and histological similarities, which can only be confirmed through immunohistochemical studies.

We herein report a challenging case of a 69-year-old female patient who presented with obstructive symptoms, diagnosed with anaplastic thyroid cancer exhibiting unusual clinical and histological features.

## Introduction

Thyroid cancer is one of the most common malignancies that predominantly affect female patients. Patients are usually asymptomatic and discovered incidentally. On the other hand, a smaller number of patients present with symptoms such as neck swelling, hoarseness of voice, or symptoms of distant metastases [1].

Thyroid malignancies are classified into differentiated and undifferentiated tumors. Anaplastic thyroid carcinoma (ATC) is an undifferentiated type of thyroid cancer that is highly aggressive and has a very poor prognosis with a median overall survival of 4-10 months [2], accounting for 2% to 3% of all thyroid neoplasms [3]. Apart from the local invasion, it frequently manifests as metastases to distant areas and regional lymph nodes [4]. However, due to its aggressive nature, patients with ATC already have metastases by the time of diagnosis, resulting in a 100% mortality rate [5].

ATC can be misdiagnosed as undifferentiated pleomorphic sarcoma (UPS), formally named malignant fibrous histiocytoma (MFH), which is the most common subtype of soft tissue sarcoma [6]. UPS is extremely rare to occur in the thyroid gland with a reported frequency ranging from 0.01% to 1.5%, with a median survival of nine months, and it has many clinical and histological similarities to ATC causing a diagnostic dilemma [7-9]. Thus, the gold standard for diagnosing UPS of the thyroid is a histopathological examination with immunohistochemical staining [10,11].

In this paper, we represent a rare case of synchronous anaplastic thyroid cancer with a foci of papillary thyroid cancer that was initially thought to be UPS.

## Case presentation

A 69-year-old female patient, known to have diabetes mellitus, morbid obesity, and a longstanding history of neck swelling for 10 years, presented to the emergency department with complaints of symptoms consistent with superior vena cava (SVC) obstruction. These symptoms included facial puffiness, blushand ing for the past three days, along with dysphagia, and dyspnea. Physical examination revealed a facial plethora with diffuse neck swelling mainly on the right side, solid in consistency, without any palpable cervical lymph nodes. There was no jugular venous distension, clubbing of fingers, or cyanosis.

The patient's vitals were normal, and labs showed WBC 7 x 10^3^/microL, hemoglobin 10.5 g/dL, platelets 315 x 10^3^/microL, International Normalized Ratio (INR) 0.9, thyroid stimulating hormone (TSH) 2.4 mU/L, and T4 7.3 mcg/dL. The echocardiogram demonstrated good left ventricular function with mild mitral regurgitation.

**Figure 1:**
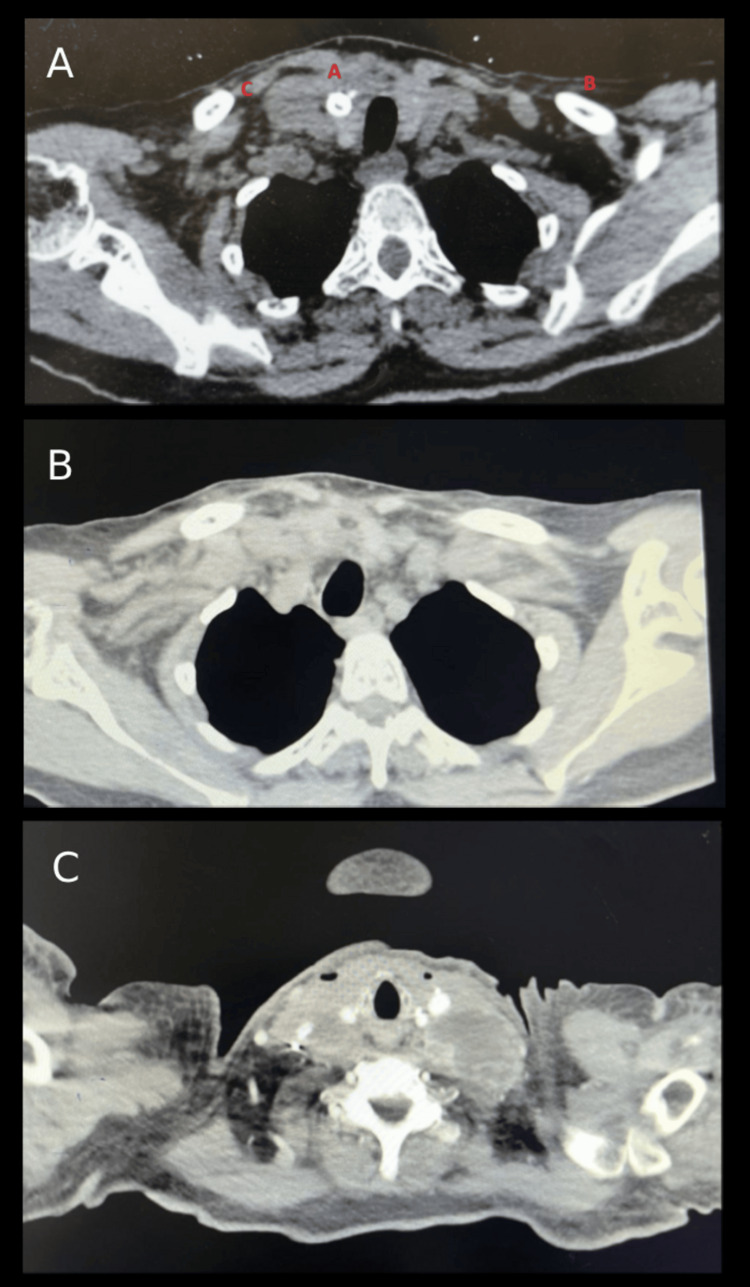
(A) Neck CT scan showing a heterogeneously enhanced thyroid gland with foci of calcification in the right lobe; (B) suspicious left supraclavicular lymph nodes, the largest measuring 13 mm x 13 mm; CT scan showing extension of thyroid into mediastinum; (C) thrombosed right internal jugular vein extending to superior vena cava; CT scan showing the postoperative hematoma.

Neck CT scan with contrast showed a heterogeneous enhanced thyroid gland with foci of calcifications in the right thyroid lobe along with multiple suspicious left supraclavicular and cervical lymph nodes as well as a thrombosed right internal jugular vein (IJV) extending into the superior vena cava (Figure 1A). Fine needle aspiration for the right thyroid revealed scattered groups and single forms of atypical cells having pleomorphic nuclei exhibiting vesicular chromatin, irregular nuclear contours, prominent nucleoli present in a background of necrosis, suppurative inflammation, and macrophages. Some of these pleomorphic nuclei also demonstrated a degree of bizarreness.

**Figure 2:**
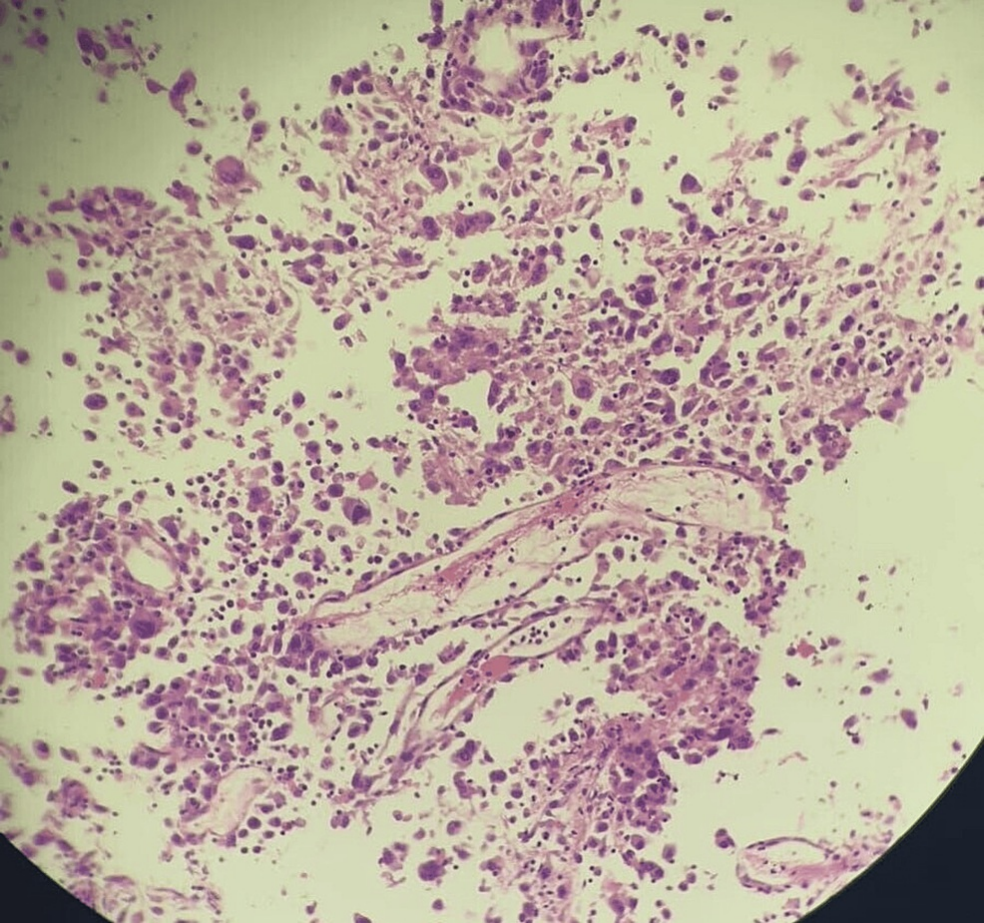
True-cut biopsy of the right thyroid nodule showing mostly necrotic tissue with a few foci of viable tissue showing a malignant spindle cell tumor with high-grade features and brisk mitotic activity (hematoxylin and eosin, magnification x10).

An ultrasound-guided true-cut biopsy of the right thyroid mass revealed a predominantly necrotic sample. There were limited viable foci showing a high-grade malignant tumor with spindle cells, consistent with the features of undifferentiated pleomorphic sarcoma (Figure 2). Another ultrasound-guided true-cut biopsy was performed on the suspicious left cervical lymph node, producing results consistent with the previous true-cut biopsy of the right thyroid nodule.

According to the biopsy results, staging was done for the patient, which involved a comprehensive assessment utilizing various imaging modalities. A neck ultrasound revealed a 15 mm x 15 mm suspicious left supraclavicular lymph node enlargement (LNE) and a suspicious 16 mm x 9 mm group IV right-sided cervical LNE. The right IJV is massively dilated and is filled with heterogenous echogenic material with mild internal vascularity within the lumen, indicative of tumoral thrombosis. Additionally, a small tubular structure extended into the right thyroid lobe from the IJV thrombus, with the anterior jugular vein thrombosed, while the external jugular vein remained patent.

Neck and chest CT with contrast revealed a heterogeneously enhanced thyroid gland with foci of calcification in the right thyroid lobe. There were multiple suspicious left supraclavicular lymph nodes, the largest measuring 13 × 13 mm, and multiple cervical nodes, with the largest measuring 9 x 14 mm, appearing necrotic beneath the right sternocleidomastoid muscle. Additionally, there were a few mediastinal lymph nodes, with the largest measuring 10 x 18 mm. The right IJV was thrombosed, extending to the superior vena cava.

Cervical MRI with contrast confirmed right thyroid lobe nodules, the largest at 1 cm x 0.8 cm, and highlighted large matted peripherally enhanced lymphadenopathy measuring about 3.9 cm x 3.2 cm x 3.1 cm at station 4 of the left deep cervical group. A few pathological lymph nodes were observed at station 4 of the right deep cervical group, with the largest measuring about 1.4 cm x 0.9 cm. Finally, a bone isotope scan yielded no evidence of bone metastasis. The findings suggest an advanced stage with involvement of regional lymph nodes and tumoral thrombosis, emphasizing the need for comprehensive management and intervention.

**Figure 3:**
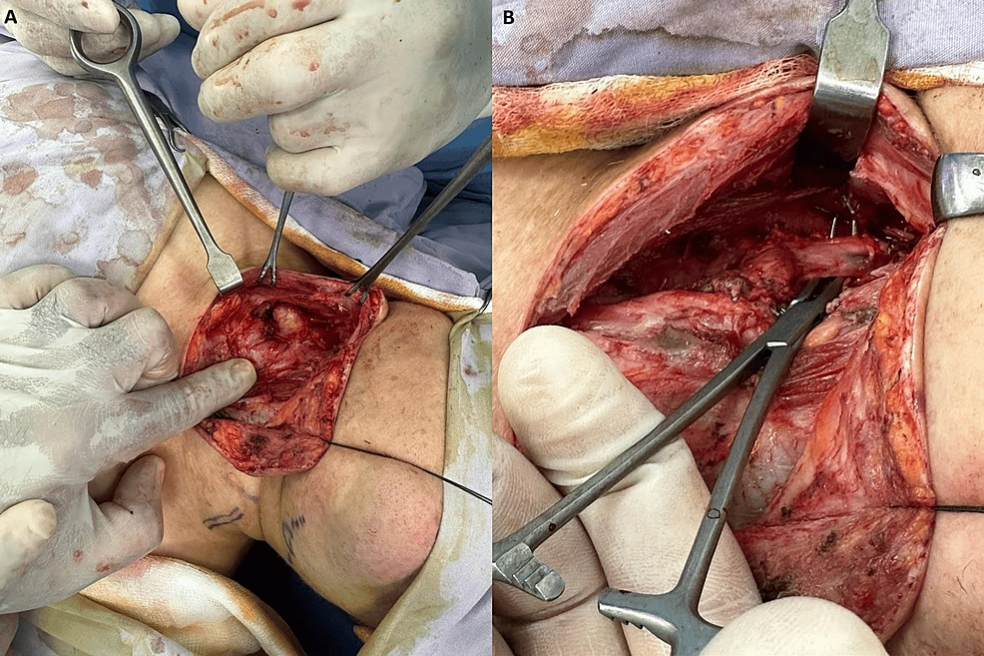
Intraoperatively showing (A) right thyroid nodule and (B) thrombosed right jugular vein.

The patient underwent total thyroidectomy and bilateral neck dissection, showing a right-sided huge thyroid mass extending to the upper mediastinum that invaded the right IJV with multiple enlarged lymph nodes bilaterally and compression of the left IJV (Figure 3). The vascular surgery team was consulted, and upon opening the lumen, tumor invasion was identified. Ligation of the right IJV was done both proximally and distally with resection of the ligated segment. On day 2 post-surgery, the patient developed neck swelling at the site of surgery and a CT scan was done which showed a 2 cm x 1 cm hematoma, which was treated conservatively (Figure 1C). The patient was then discharged on day 7 to be followed up at the clinic.

**Figure 4:**
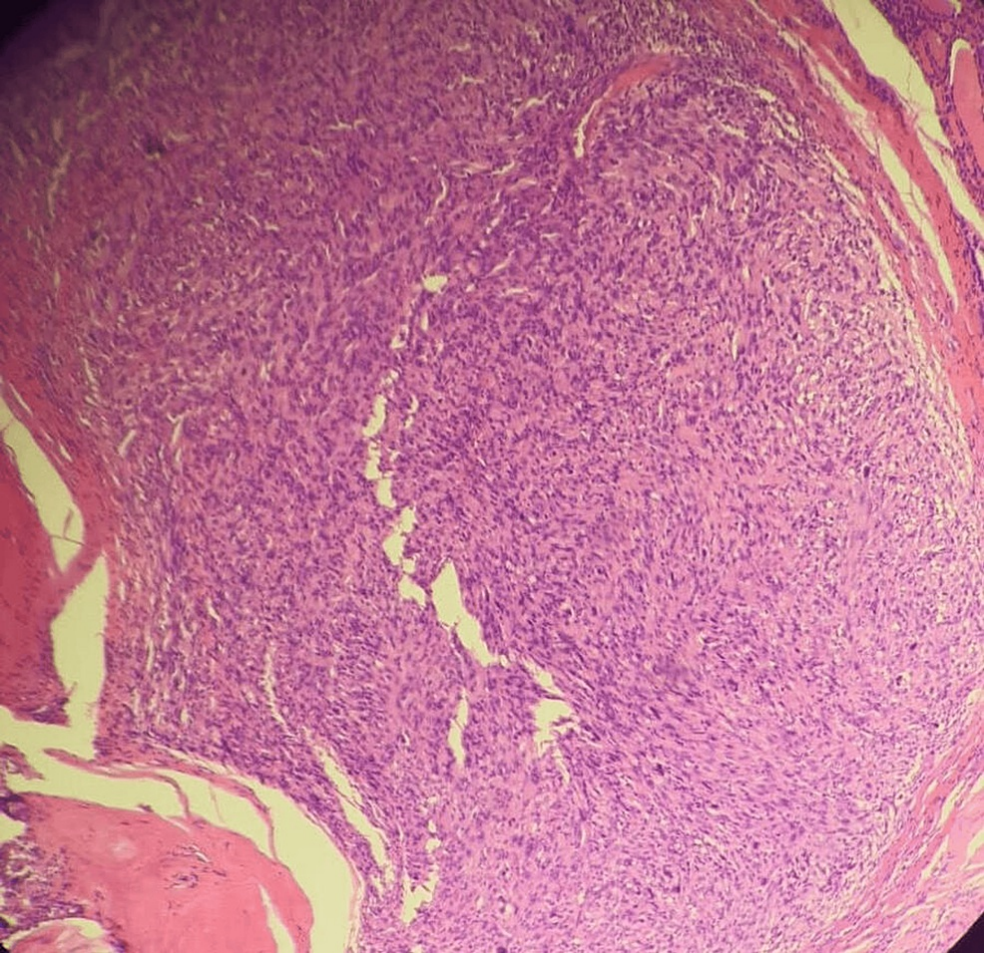
Microscopic examination of the nodule revealed an anaplastic thyroid carcinoma with adjacent small foci of papillary thyroid carcinoma (hematoxylin and eosin, magnification x20).

**Figure 5:**
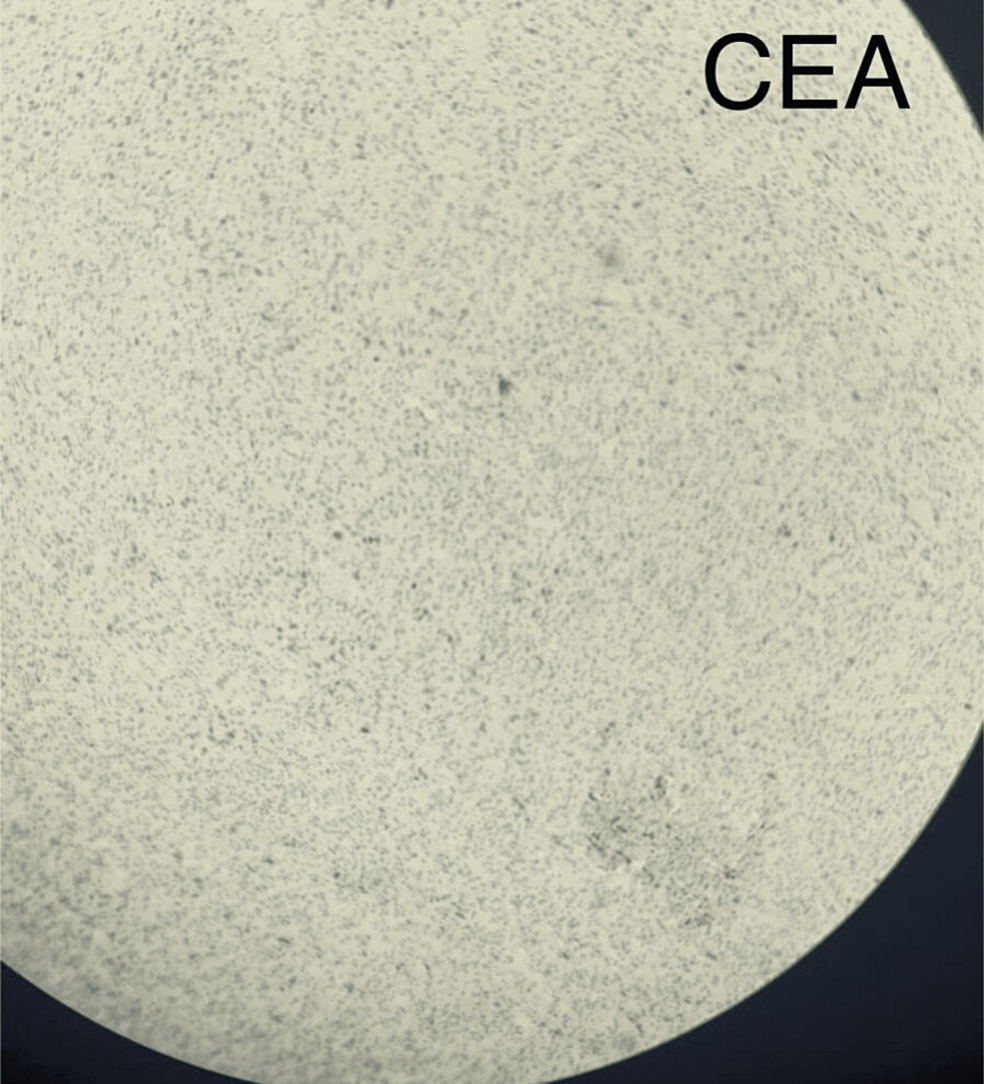
Immunohistochemical staining of thyroid tissue showing focal positivity for cytokeratin 7 (CK7), cytokeratin 8/18 (CK8/18), and cell adhesion molecule 5.2 (CAM5.2), while thyroglobulin, paired box gene 8 (PAX8), calcitonin, thyroid transcription factor-1 (TTF-1), epithelial membrane antigen (EMA), and carcinoembryonic antigen (CEA) being negative (magnification x10).

Microscopic examination of the thyroid specimen revealed an ATC with adjacent small foci of papillary thyroid carcinoma with lymphovascular and perineural invasion and positive resection margins (Figure 4). Immunohistochemical stains were positive for vimentin, cytokeratin 7 (CK7), CK8, CK18, and cell adhesion molecule 5.2 (CAM5.2) and negative for thyroglobulin, calcitonin, carcinoembryonic antigen (CEA), epithelial membrane antigen (EMA), thyroid transcription factor-1 (TTF-1), and paired box gene 8 (PAX8) (Figure 5). 

The case was then discussed in a multidisciplinary team meeting, involving a radiologist, endocrine surgeon, endocrinologist, oncologist, and histopathologist. The treatment plan was to start the patient on adjuvant chemoradiotherapy, which included adjuvant concurrent external beam radiation therapy (five sessions per week) with radiosensitizing chemotherapy (one session per week) for six weeks. The chemotherapy included Docetaxel 20 mg/m^2^ intravenous (IV) and doxorubicin 20 mg/m^2^ IV, alongside a radiation dose of 60 Gy. Unfortunately, at the third session of chemotherapy, the patient had chest pain and shortness of breath. A pulmonary CT angiography revealed a pulmonary embolism, and the patient was started on heparin infusion. The patient was transferred to the intensive care unit (ICU), and the following day, she developed respiratory arrest and passed away.

## Discussion

Anaplastic thyroid cancer is a rare but highly aggressive form of thyroid carcinoma, exhibiting the poorest prognosis among all thyroid cancers, with a median survival of five months. Comprising less than 2% of all thyroid cancers, it yet accounts for 40% of deaths related to thyroid cancers [12]. Factors associated with an increased risk of developing ATC include preexisting thyroid pathology (e.g., long history of goiter, follicular thyroid cancer, and papillary thyroid cancer), female gender, low education level, and old age [13,14]. The majority of ATCs originate from differentiated thyroid cancers, with papillary thyroid carcinoma being the most common [15].

SVC obstruction is most commonly secondary to bronchogenic carcinoma or lymphoma but seldom rare and could be due to advanced thyroid neoplasm such as ATC by exponential growth through the mediastinum and compression of the structures causing thrombosis of the venous system. Although SVC is associated with advanced thyroid disease, it can still occur in the absence of an obvious mass in the thyroid gland [16,17].

According to the 2021 American Thyroid Association (ATA) guidelines, the recommended treatment for ATC still includes thyroidectomy if the tumor is resectable, along with chemoradiation. However, most ATCs are nonresectable due to the invasion of cervical structures. Additionally, newer treatments are emerging, such as targeted therapy based on the patient's genetic profile; however, survival rates remain low [18,19].

Unlike differentiated thyroid carcinoma, ATC is very resistant to conventional chemotherapy and radiotherapy; thus, there has been an increase in the development of targeted therapies. The most common mutation in ATC is BRAF (V600E). Dabrafenib plus Trametinib (DT) are BRAF and MEK inhibitors that have gained approval for treating advanced solid tumors with the BRAF (V600E) mutation, demonstrating a notable response rate of over 50% in ATC [20,21]. 

Larotrectinib is a tropomyosin receptor kinase (TRK) inhibitor that has been approved for patients with advanced solid tumors with neurotrophic receptor tyrosine kinase (NTRK) fusion. In ATC, larotrectinib demonstrated a response rate of 29%, exceeding that of conventional chemotherapy [18,21,22]. 

Lenvatinib, an anti-angiogenic agent approved for radioiodine refractory thyroid cancers, has shown a controversial role in ATCs. A single-arm phase II trial showed a median progression-free survival benefit of 7.4 months, a median overall survival of 10.6 months, and an overall response rate of 24% [23]. However, a recent phase II trial was stopped due to ineffectiveness as the preliminary analysis showed a very low response rate (2.9%) and survival outcome [24]. 

Another rare variant of thyroid carcinoma is undifferentiated pleomorphic sarcoma of the thyroid (UPS-T), previously named MFH, which is very challenging to differentiate from anaplastic thyroid cancer as both entities may present as rapidly growing thyroid mass in elderly patients to even obstructive symptoms. Moreover, both are characterized by the diffuse infiltration of lymphoid cells and the presence of spindle cells, pleomorphism, and a storiform pattern. However, fine needle aspiration cytology and ultrasonographic features do not help differentiate those tumors. 

To differentiate between them, a combination of histopathologic morphology and immunohistochemical stains is required. Both are positive for vimentin, but only ATC is positive for cytokeratin and only UPS-T is positive for positive for α1-antitrypsin and CD68 [11,25]. In our case, the diagnosis of ATC was ultimately confirmed based on histological features and immunohistochemical results. A comparison between the characteristics of ATC and UPS-T is shown in Table 1.

**Table 1:** Comparison between anaplastic thyroid carcinoma and undifferentiated pleomorphic sarcoma of the thyroid. LN, lymph node; (‐), negative; (+), positive; UPS, undifferentiated pleomorphic sarcoma

Characteristics	Anaplastic thyroid carcinoma	UPS‐Thyroid
Incidence	<2%	0.01%-1.5%
Patient population	Elderly	Elderly
Symptoms	Compressive	Compressive
Regional LN metastases	Common	Uncommon
Histopathologic features	Spindle cell, pleomorphism, and storiform pattern	
Immunohistochemistry		
Vimentin	+	+
CD68	-	+
CK	+	-
α1‐Antitrypsin	-	+

The preferred treatment for UPS-T is complete excision, with or without subsequent adjuvant radiotherapy and/or chemotherapy; however, the role of the adjuvant treatment remains debatable as there is no clear trend indicating a positive impact on survival [11]. In contrast, a study conducted by Huber et al. suggested a potential benefit of adjuvant radiotherapy [26].

## Conclusions

In conclusion, the clinical presentation of ATC varies from a solitary neck mass to symptoms of mass effect or/and local invasion, such as hoarseness of voice, dysphagia, dyspnea, as well as symptoms of SVC obstruction. However, the diagnosis of anaplastic thyroid cancer remains challenging as it is similar to UPS-T. Thus, histopathologic features and immunohistochemical stains are required for diagnosis. The treatment of choice for ATC is debulking surgery with or without chemoradiotherapy.
